# The long and short of it: travel distance and territorial intruder pressure predict central-place spawning tactics among Caribbean *Stegastes* damselfishes

**DOI:** 10.1007/s00442-025-05761-w

**Published:** 2025-07-08

**Authors:** Taylor L. Hobbs, Richard S. Nemeth, Donna Nemeth, Kayla M. Blincow, Paul C. Sikkel

**Affiliations:** 1 Center for Marine and Environmental Studies, University of the Virgin Islands, United States Virgin Islands, St. Thomas, 00802 USA; 2 Department of Marine Biology and Ecology, Rosenstiel School of Marine, Atmospheric and Earth Sciences University of Miami, Miami, FL USA; 3 Water Research Group, Unit for Environmental Sciences and Management, North-West University, Potchefstroom, South Africa

**Keywords:** Central place activities, Fish reproduction, Marine ecology, Behavioral ecology, Territoriality, Coral reefs, Decision-making, Conflicting demands, Tradeoffs, Parental care

## Abstract

Central place activities present conflicting demands or tradeoffs between activities at and away from the central place. *Stegastes* damselfishes are small, mostly reef-associated fishes that are common in shallow tropical and sub-tropical marine environments. Both sexes defend a territory consisting of their food supply and shelter sites, with male territories also including a nest where females lay eggs which are then guarded by the male. Females therefore need to leave their territory to spawn in male nests, putting them at risk of attack during travel and leaving their territory exposed to intruders while away. Female egg-laying tactics can vary among and within species—some complete spawning during a single, extended bout, while others do so in multiple bouts, separated by visits to their territory. Spawning behavior and behaviors associated with territoriality of 101 individuals from 6 species were observed between June 2022 and May 2023 to identify and quantify the factors that influence variation in spawning tactics both within and among *Stegastes* species. Specifically, we tested two predictions of the hypothesis that dividing spawning with a single male into bouts allows individuals to balance the tradeoff between travel costs and intruder pressure for females of permanently territorial damselfishes: (1) intruder pressure will be positively related to the number of spawning trips among species and/or among females within species; and (2) travel distance will be negatively related to the number of spawning trips. Results of this study showed that *Stegastes* females increase their spawning bouts with an increase in intruder pressure and decrease their spawning bouts with an increase in travel distance.

**Supplementary Information:**

The online version contains supplementary material available at 10.1007/s00442-025-05761-w.

## Introduction

Among the myriads of ecological pressures faced by free-living organisms are fitness-consequential decisions involving mate choice, enemy avoidance, diet selection, habitat selection, migratory strategies, and home range size (Mcfarland 1977, Dill 1987, Dukas 1998, Davies et al. 2018, Martin and Bateson 2018). Since organisms rarely face a single decision or environmental challenge, adaptive decisions typically involve tradeoffs between two or more conflicting demands. For example, increased foraging effort may result in an increased risk of predation or parasitism (Lewis et al. 2004; Steele et al. 2014, Gabel et al. 2023). While a home range likely encompasses all an individual’s necessary resources, many species have a specific central place that is a subset of that home range. This central place includes one or more of their essential resources, such as a nesting site or feeding territory. However, because this place likely does not include all vital resources, organisms will need to venture away from their central place to obtain other resources or complete fitness-enhancing tasks, creating a “central place effect” (Olsson et al. 2008; Stephens et al. 2008).

Central place effects, and the ways in which animals solve them, have been studied mostly in birds (Bryant and Turner 1982; Kacelnik 1984; Lewis et al. 2004; Burke and Montevecchi 2009; Ainley and Ballard 2012), but also mammals (Houston and McNamara 1985; Newman et al. 1988; Beauplet et al. 2004; Steele et al. 2014; Friedlaender et al. 2016, Thometz 2016) and even some fishes (Tricas 1989; Warner and Schultz 1992; Karino and Kuwamura 1997; Sikkel 1998; Sikkel and Kramer 2006). The most widely studied central-place effect is central-place foraging (CPF) (Orians and Pearson 1979; Bell 1990; Olsson et al. 2008; Burke and Montevecchi 2009) and has provided a solid theoretical framework from which to study central-place effects more broadly. According to CPF theory, optimal foraging strategies serve to maximize an organism’s net energetic gain given the tradeoffs imposed by the need to forage away from the central place. Organisms foraging according to predictions of CPF theory should, therefore, maximize the rate of harvest or energetic gain per unit of time, while balancing associated costs, such as missed opportunities, predation risk, and the energetic costs associated with travel. These predictions from CPF theory can then be generalized to other behaviors, such as air breathing in aquatic animals (Kramer 1988; Heath et al. 2007). Broadly, for organisms faced with central-place effects, selection is expected to favor behaviors that most effectively manage all system-specific tradeoffs, maximizing net benefits.

The comparative method has been widely used in behavioral ecology to understand the costs and benefits and selective pressures shaping animal behavior (Eibl-Eibesfeldt and Kramer 1958; Réale et al. 2007; Hailman 2019; Bolhuis et al. 2021). By comparing multiple organisms that exhibit similar behaviors and face similar challenges, it is possible to arrive at a more comprehensive theory of central-place activities. However, as with most other areas of behavioral ecology, empirical studies on vertebrates are biased heavily toward birds and mammals and those studies related to central-place activities focus primarily on foraging. Thus, by broadening both the taxonomic coverage and types of central-place activities, we stand to generate a more comprehensive theoretical framework.

Many common central-place activities in fishes are related to territoriality, a common behavioral trait in fishes, especially coral-reef species (Colin 1971; Tricas 1989; Clifton 1990; Warner and Schultz 1992; Cowlishaw 2014). Defense of a territory is usually associated with defending one or more resources, such as food, shelter, and spawning sites (Clifton 1990; Sikkel 1998, Ah‐King et al. 2005). A central-place territory allows the individual to maintain those essential resources but still typically requires them to leave in search of other resources existing outside their territory. Defense of a resource has costs, such as a risk of injury or energy expenditure, as well as benefits, such as priority access to the protected resource (Kaufmann 1983). Many fish species defend a territory to prioritize or use a particular resource. A few examples are the bluehead wrasse (*Thalassoma bifasciatum*) where some males use territoriality as a mating tactic (Warner and Schultz 1992), corallivorous butterflyfish (*Chaetodon multicinctus)* which utilize a territory as a feeding ground *(*Tricas 1989*),* and herbivorous striped parrotfish (*Scarus iserti)* where females will co-defend feeding territories (Clifton 1990).

Territoriality in fishes is often directly related to parental care (Reynolds et al. 2002; Davies et al. 2018). While in most fish species, there is no parental care for the offspring, in species that that do provide parental care, it is typically provided solely by the male (Gross and Sargent 1985, Benun and Wison 2019). Because the female typically lays her eggs in the male’s territory (Gross and Sargent 1985, Ah‐King et al. 2005, Davies et al. 2018), females must visit the male’s territory to spawn.

Damselfishes (family Pomacentridae) include over 300 species of small (< 30 cm) fishes. Most live in shallow, clear, tropical water, but there are some warm-temperate species and species that occur in deeper water (Allen 1991). Apart from some planktivorous species such as those in the genus *Chromis*, most damselfish species live in close association with the benthos, feed from the substrate, and lay benthic eggs. Males of all species (except the bi-parental *Acanthochromis*) defend a territory during spawning. However, in many species, both sexes defend multi-purpose territories that include a food source (often turf algae), shelter sites, and for males, nest sites (Sikkel 1998; Sikkel and Kramer 2006; Ceccarelli 2007). In such damselfish species, spawning typically takes place at dawn in various months throughout the year, depending on species and location (Kohda 1988; Robertson 1990; Danilowicz 1995; Tzioumis and Kingsford 1995). Like many marine organisms, reproductive activity is linked to the lunar cycle, typically resulting in a monthly peak (Gladstone and Westoby 1988; Robertson 1990, 1991). Before spawning takes place, females will search for a mate and males will display courting behavior to attract the females’ attention. For dawn-spawning species, mate choice decisions are in most cases made the day prior to spawning and are thus temporally decoupled from the spawning event. At the first light, females then leave their territory and swim directly to the male they have chosen and typically spawn with only that male on a given day. Once she reaches the male’s territory, she will lay a clutch of adhesive eggs. Egg-laying alone requires up to 100 min, depending on female body size and species. The male then cares for the eggs until they hatch (Karino 1995a; Karino 1995b; Reynolds et al. 2002). Because the female must leave her territory to spawn, females of permanently territorial damselfish are an ideal model system for the study of central-place problem-solving.

Permanently territorial damselfish have a territory that includes a food supply which could be ‘raided’. Thus, as for a territorial central-place forager, leaving the territory to spawn could make it vulnerable to damage or loss, and travel to and from the male nest would result in energetic expenditure as well as possible risk of injury or death (Houston and McNamara 1985; Steele et al. 2014, Thometz 2016). In particular, heterospecific intruders could capitalize on the resident’s algal food source and conspecifics could attempt to take over part or all of the territory while the female is engaged in spawning. Since females must traverse the territories of other damselfishes en-route to the male territory, they risk being attacked by both predators and conspecifics during travel (Karino and Kuwamura 1997). While these risks parallel the principles of CPF theory, in the case of damselfish, rather than accruing an energetic resource while away from the central place, individuals must balance the benefits of laying eggs in a single nest, and having them cared for, against the risks of leaving their territory undefended and costs associated with travel between their territory and the male nest.

As a possible solution to this tradeoff, females can allocate their egg-laying effort over multiple short bouts, returning to their territory between bouts rather than simply leaving their territory to spawn in a single prolonged event, and returning when finished (Karino and Kuwamura 1997; Sikkel 1998; Sikkel and Kramer 2006). It is unknown to what extent this variation in spawning tactics occurs within or among species, and precisely which ecological variables influence female decisions.

Dividing spawning into bouts may allow individuals to balance the tradeoff between travel costs and intruder pressure for females of permanently territorial damselfish; however, only three studies have thus far been conducted on this topic, each with a different focus. Karino and Kuwamura (1997) focused on the role of mate proximity in determining the allocation of spawning trips. Sikkel (1998) focused on the role of intruder pressure in mate-searching tactics, and Sikkel and Kramer (2006) on how revisits to the territory during spawning effectively reduced intruder pressure. Combined, these studies included only three species, from three different genera, and were conducted in three different parts of the world. There are other potential costs and benefits that were not considered in these studies. In this study, we further consider the central-place problem of egg laying (spawning) by female damselfish, once a male nest has been chosen. We take advantage of the presence of multiple sympatric and closely related (congeneric) species of permanently territorial damselfishes that differ in physical and habitat variables that can impact the opposing costs of travel and being away from the territory. We use intra- and interspecific comparisons of these species to test predictions that (1) intruder pressure will be positively related to the number of spawning trips among species and/or among females within species; and (2) travel distance will be negatively related to the number of spawning trips.

## Methods

### Study species

All *Stegastes* species in the Caribbean region inhabit inshore reef or rocky/rubble reef environments, and both sexes defend a territory consisting of their food supply and shelter sites. As in other permanently territorial damselfishes, the males defend dual-purpose feeding and spawning territories. Habitat characteristics and lunar spawning periodicity vary slightly among species (Waldner and Robertson 1980; Robertson et al 1990). However, spawning for all species begins at first light and is completed shortly after sunrise (Kohda 1988; Robertson 1990). The process of mate assessment by females occurs during the several days prior to the spawning event, with the female swimming directly to the nest of the chosen male at first light and then laying all her eggs for that day in the nest of a single male (Knapp and Kovach 1991; Petersen 1995). Egg laying alone in these species requires up to 52 min and can be completed in a single event or divided into multiple, shorter bouts but which will prolong the overall process. This project focused on the six species of *Stegastes* found in the United States Virgin Islands (USVI) (Fig. S1). These include *S. adustus* (dusky damselfish), *S. variabilis* (cocoa damselfish), *S. planifrons* (three spot damselfish), *S. leucostictus* (beaugregory damselfish), *S. partitus* (bicolor damselfish), and *S. diencaeus* (longfin damselfish).

### Study sites

We selected the sites (Fig. S2) based on the presence of *Stegastes* species and ease of access. All six target species were prevalent along the shorelines of the US Virgin Islands (USVI), and all sites were accessible by shore. We selected at least one site on each of the three United States Virgin Islands (St. Thomas, St. John, St. Croix), with St. Thomas having the most sites. We accessed sites via snorkel or scuba with a maximum depth of 11.3 m which included: St. Thomas: Brewers Reef (coral reef, N 18.34311, W -64.98220), Range Cay (rocky/rubble reef, N 18.33995, W -64.97727), Black Point (boulder reef, N 18.34560, W -64.98499,), Lindbergh Bay (rocky reef, N 18.33180, W -64.96433); St. John: Lameshur Bay (rocky and coral reef, N 18.31720, W -64.72424); St. Croix: Cane Bay (coral reef, N 17.77323, W -64.81119).

### Behavioral observations

As *Stegastes* in the study region spawn over a period of about 8 months (March–October), data collection occurred June–October 2022 and March–April 2023. We observed species at different times throughout the month according to their species-specific lunar spawning schedules (Robertson et al. 1990).

We observed each of a total of 101 females twice, totaling 202 observations (Table S1), once during spawning, and once on a non-spawning day during the same time of day to estimate the degree of territoriality during the spawning period. For spawning observations, an observer entered the water 30 min before sunrise with a clipboard, waterproof paper, pencil, timing device, and light. The observations started when a male and female were found in the same nest (Fig. S3). We then recorded arrival and departure times (i.e., arrival to male’s territory, departure from male’s territory, arrival at females’ territory, and departure from females’ territory) to the nearest second throughout the spawning session. Each time the female visited the male and spawned was considered one bout.

We recorded attacks from other damselfish that occurred while the female was traveling between her territory and the male’s territory. We also recorded defense of the territory while the female was “home”. Each time, the individual female rushed or chased another fish was considered a defensive attack. The observations were concluded when the individual female had not left her territory for 20–30 min.

Once the spawning period concluded, we placed flagging tape in both female and male territories, color-coded by sex. This was done to verify the location when returning the following day to conduct observations on female territoriality. The depth of the female’s territory was recorded using a dive computer to the nearest 0.1 m. We estimated female body size using a pencil as a scale, approaching as close as possible to the fish to measure its length which was then marked on the datasheet and measured using a ruler to the nearest mm.

Because *Stegastes* females do not spawn on consecutive days, for each female, we conducted the second focal animal observation the morning following the spawning observation (thus ensuring that the individual would not be spawning at the time of the observation). If conditions did not allow the “territoriality” observation to occur the following day, we completed the observation during the same time of day on another non-spawning day within 3 days of the spawning observation. For each “territoriality” observation, we began within 5 min of the start time of the individual’s previously recorded spawning session and recorded activity for a total of 1 h, divided into six 10 min segments. We used the same methods for recording defensive behaviors as used in spawning observations. For the purpose of analysis, we converted territoriality data to chases per hour, or “chase rates”.

In cases where multiple females needed to be watched at once, the territoriality data were collected electronically through the placement of GoPro cameras. In these cases, we set two cameras on either side of the territory facing each other. They were placed on adjustable arms and then attached to a weight to withstand mild surge. Recording began in the morning, 5 min before the individual’s previously recorded spawning start time. After an hour, they were collected and reviewed in the lab. The same behaviors and feeding bouts were recorded from the videos as the in-person observations.

### Characterization of habitat

We placed quadrats at each of the female territories after both spawning and territoriality observations had taken place (see *Behavioral Observations* above). A standard size of 1 × 1 m quadrat was used for each fish regardless of territory size. We placed the center of the quadrat in the center of the individual territories. We measured rugosity in two directions perpendicular to each other using a transect tape allowing it to rise and fall with the topography of the reef and the total length of the line was recorded. We then divided the distance by the total length to obtain a rugosity measurement between 1 and 0 where 1 was flat and 0 was rugose (Risk 1972). The benthic composition (e.g., live coral, boulder, rock, rubble, and sand) within the quadrat was estimated and recorded as percent cover.

### Statistical analyses

The combination of behavioral and environmental data recorded is summarized in Table 1. All assumptions were met, and all tests and statistical analyses were performed using R version 4.4.0 (R Core Team 2024). We used Pearson correlation tests to determine: (1) The association between the amount of time a female spent spawning (spawning time) and time (minutes) from sunrise; (2) The amount of time a female spent at home within her territory during spawning (home time) and time from sunrise; (3) The association between territoriality and time from sunrise; and (4) The association between the total duration of a spawning session (including home time, spawning time, and travel time) and number of bouts in a spawning session. The result of this latter correlation showed that there was a strong association between total time and bouts (*r*(99) = 0.65, *p* < 0.001). To account for variability in the total time across different spawning sessions and thereby variability in the number of spawning bouts, bouts per minute (bpm) was used as the dependent variable in further analyses. Finally, (5) We performed Pearson correlation tests to determine the association between territoriality and travel distance between mating pairs (distance) across all species and for each species individually.

**Table 1:** List of variables included in this study

Variable	Description	Unit
Biological		
Species	Each of the six *Stegastes* species in the USVI	–
Female body size	Estimation of total length of each fish	Millimeters
Distance	Distance of female territory from nest	Meters
Territoriality	Rate of chases made by a female in her territory during spawning time but on non-spawning days	Chases/hr^−1^
Defensive attack	Number of chases made by a female in her territory during spawning	Chases/bout
Dependent		
Total time	Total time from start of observation to end including time female spent traveling, defending, and spawning	Seconds
Spawning bouts	The number of times a female returned to her territory in a spawning session	Bouts/min^−1^
Environmental		
Rugosity	Measurements of benthic structure from individual territories	100 cm/100 cm + number
Benthic habitat	Benthic habitat composition of female territories	Percentage of cover
Sunrise	The time of sunrise	Hours/min

We used linear models to test for the influence of the biological variables (female body size, distance, and territoriality) on bpm across all *Stegastes* species and for each species individually. To avoid issues associated with collinearity, we conducted correlation analyses to identify any instances of high correlation between each of the explanatory variables. Body size was highly correlated with distance and moderately correlated with territoriality, and thus, it was removed from subsequent modeling.

We performed Analysis of Variance (ANOVA) tests and corresponding Bonferroni post hoc tests to test for differences in benthic cover of home territories (i.e., boulder, coral, sand, and rubble) across *Stegastes* species. We performed additional ANOVAs to test differences in the biological and behavioral variables (i.e., body size, travel speed, bouts per minute, territoriality, and distance) between species. A Pearson correlation test was performed to determine the association between spawning time and number of defensive attacks during spawning. We used a principal component analysis (PCA) to visualize and identify multivariate patterns across species based on the following variables: bpm, territoriality, travel speed, body size, and territory rugosity. The PCA was done using the vegan package version 2.6–6.1 (Oksanen 2024).

To assess the potential contribution of phylogenetic relatedness to interspecific differences in spawning trip frequency, we superimposed phylogenetic position within Caribbean *Stegastes* from Tang et al. (2021) onto the results of the PCA.

## Results

Spawning sessions varied greatly in both time and number of bouts across species and individuals. Spawning variables and female traits, including distance traveled to spawn, territoriality, and bpm all differed among species, as did most habitat variables (Table S2).

### Spawning bouts relative to time of day

The number of bouts made by female *Stegastes* during spawning hours peaked within 5 min of sunrise and decreased throughout the spawning session. Peak bout times varied by species (Fig. S4). *S. adustus, S. planifrons,* and *S. diencaeus* all peaked around sunrise, while *S. partitus, S. leucostictus*, and *S. variabilis* had less-defined peak bout times.

For all *Stegastes,* there was a significant negative association between the time from sunrise and spawning time, *r*(656) = − 0.35, *p* < 0.001 and there was a non-significant positive association between time from sunrise and time in the home territory, *r*(554) = 0.03, *p* = 0.50. The strength of the correlation between time from sunrise and both spawning time and time in home territory varied by species (Fig. S5, Table 2). Territoriality was positively correlated with the time from sunrise and the strength of the correlations varied by species (Table 3).

**Table 2:** Pearson correlation between home time (time spent in the home territory during a spawning session) (left) and spawning time (time spent spawning during a spawning session) (right) in relation to time from sunrise for each of the six study species

Species	Home time			Spawning time		
	*r*	*p*	Correlation strength	*r*	*p*	Correlation strength
*S. partitus*	− 0.01	0.947	Medium	− 0.53	< 0.001	High
*S. planifrons*	0.31	0.001	Medium	− 0.41	< 0.001	Medium
*S. variabilis*	0.16	0.519	Low	− 0.63	< 0.001	High
*S. adustus*	0.09	0.149	Low	− 0.28	< 0.001	Low
*S. diencaeus*	− 0.1	0.34	Low	− 0.49	< 0.001	Medium
*S. leucostictus*	− 0.34	0.239	Medium	− 0.63	0.005	High

**Table 3:** Correlation between chase rates and the time from sunrise for each of the six study species

Species	df	*r*	*p*
*S. partitus*	112	0.19	0.038
*S. planifrons*	112	0.42	< 0.001
*S. adustus*	82	0.47	< 0.001
*S. diencaeus*	88	0.39	< 0.001
*S. leucostictus*	100	0.14	0.15
*S. variabilis*	52	0.3	0.027

### Effects of biological variables on spawning bouts

**Fig. 1:**
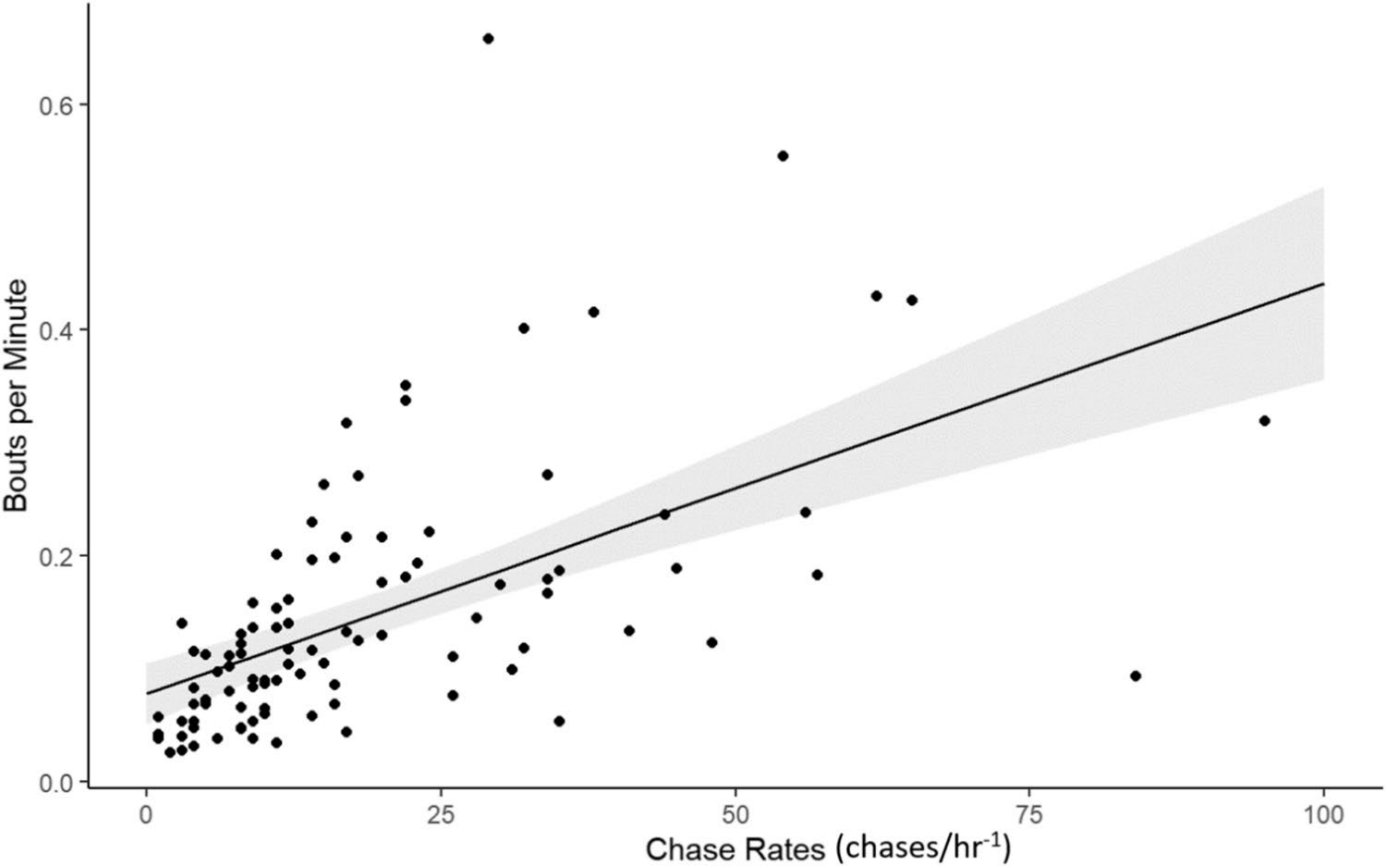
The relationship between bouts per minute (bpm) and chase rates for six species of damselfish showing raw data (black dots), the best fit line based on our linear regression model (black line), and the 95% confidence interval (gray shaded area) (*N* = 101)

**Fig. 2:**
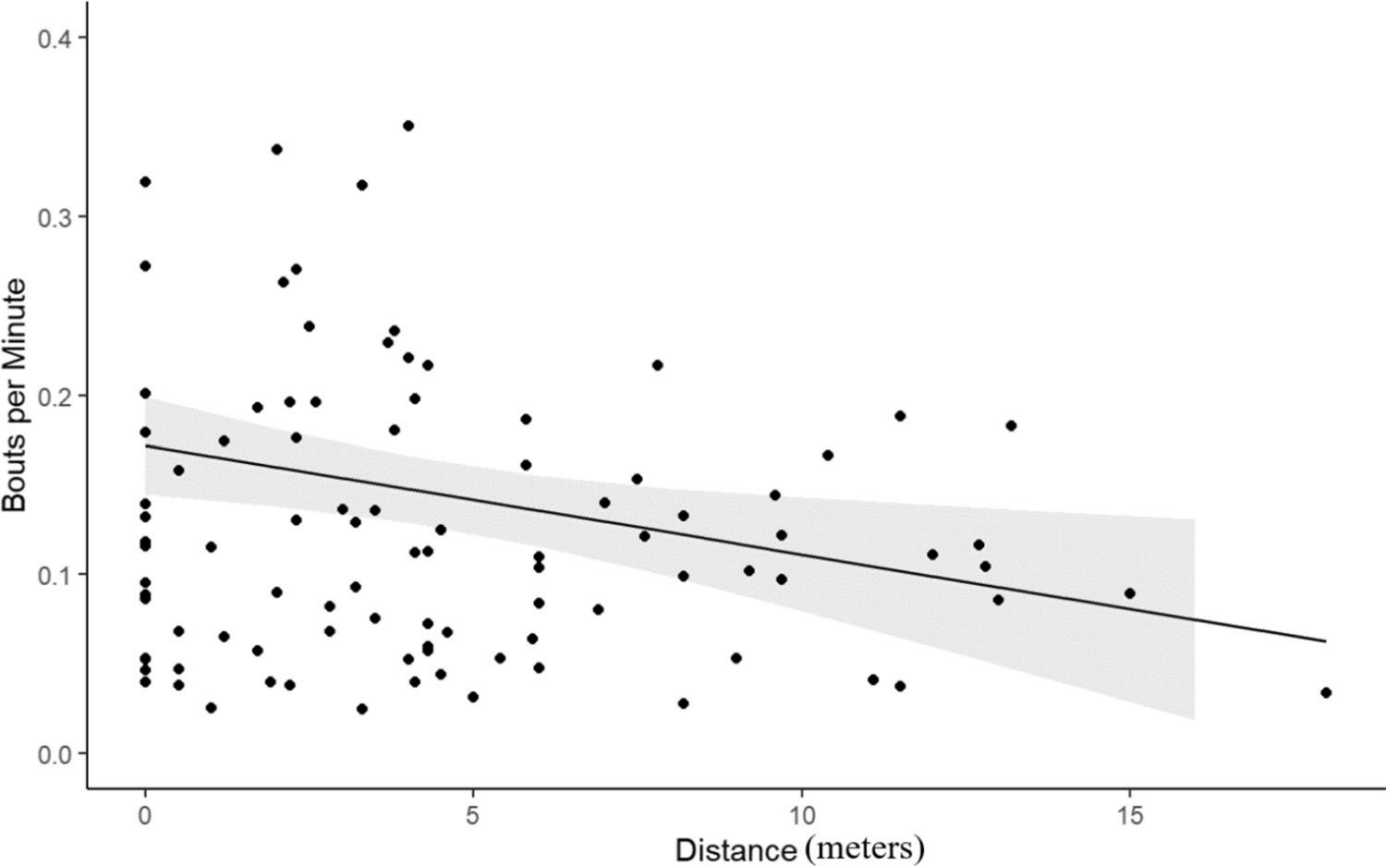
The relationship between bouts per minute and distance for six species of damselfish using the predicted values (all species combined) of bouts per minute in relation to distance. This shows raw data (black dots), the best fit line based on our linear regression model (black line), and the 95% confidence interval (gray shaded area) (*N* = 101)

The model relating bpm to the biological variables (distance and territoriality) across all species was statistically significant (*R*^2^ = 0.36, *F* = 27.84, *p* = 1.783e-10). The model indicated a significant effect of both territoriality (*β* = 0.003, *p* < 0.001); Fig. 1) and travel distance (*β* = − 0.006, *p* = 0.01; Fig. 2).

**Fig. 3:**
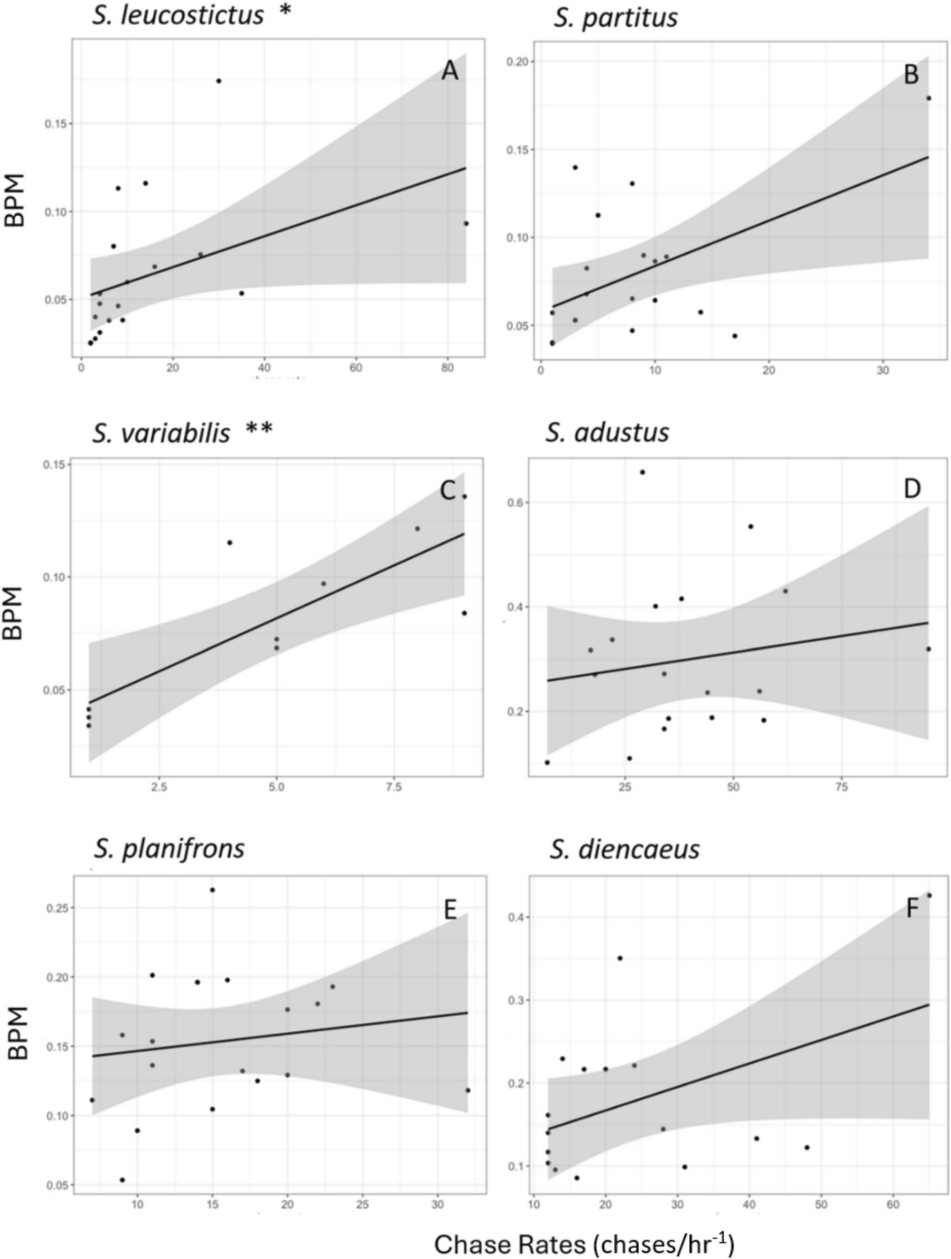
Relationship between bpm and territoriality for each species (**A**–**F**). This shows significant relationships (asterisks), raw data (black dots), the best fit line based on our linear regression model (black line), and the 95% confidence interval of that relationship (gray shaded area). Note the differences in the axis scales for each species (N_A_ = 19; N_B_ = 19; N_C_ = 10; N_D_ = 18; N_E_ = 19; N_F_ = 16)

**Fig. 4:**
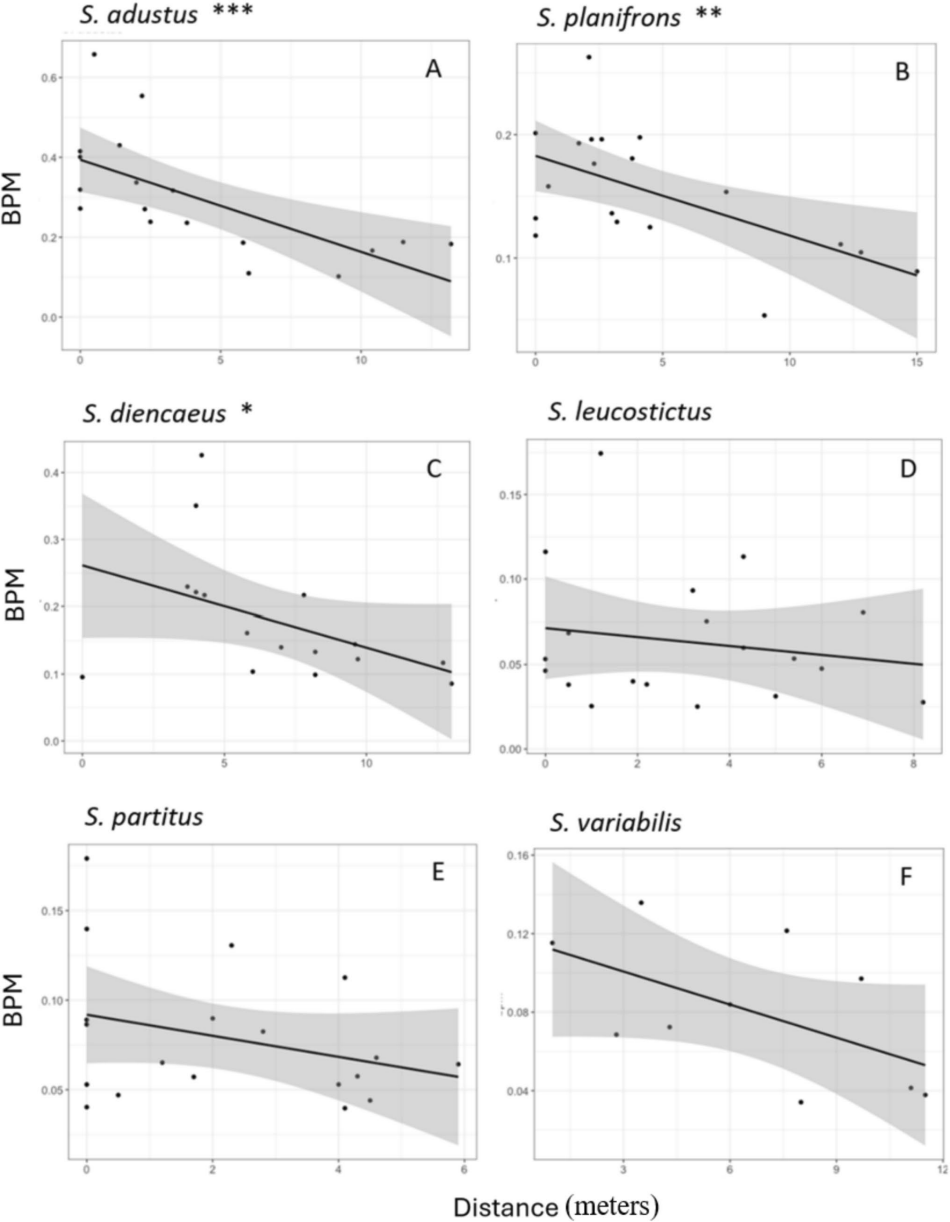
Relationship between bpm and distance for each species (**A**–**F**). This shows significant relationships (asterisks), raw data (black dots), the best fit line based on our linear regression model (black line), and the 95% confidence interval of that relationship (gray shaded area). Note the differences in the axis scales for each species (N_A_ = 18; N_B_ = 19; N_C_ = 16; N_D_ = 19; N_E_ = 19; N_F_ = 10)

Results for the species-specific model formulations varied in terms of statistical significance (Table 4). Territoriality was a significant predictor of bpm for *S. variabilis* and *S. leucostictus* (Table 4, Fig. 3). The *S. partitus* model showed that territoriality was more influential than distance, but it was not significant (Table 4, Fig. 3). Distance was a significant predictor of bpm for *S. adustus, S. planifrons,* and *S. diencaeus* (Table 4, Fig. 4). Results showed that there was a positive correlation of bpm and territoriality for all six species and a negative correlation of bpm and distance for all six species (Table 5).

**Table 4:** Results of linear model relating bpm to the biological variables (distance and territoriality) for each species including degrees of freedom (DF), adjusted *R*^2^ (ADJ *R*^2^), F-statistic, overall model significance (*p* value), significant variables, slope estimates for significant variables, and variable specific *p* values (Pr( >|t|))

	DF	ADJ R^2^	*F* value	*p* value	Significant variable	Slope estimate	Pr( >|t|)
*S. adustus*	15	0.49	9.29	0.002	Distance	− 0.083	0.001
*S. partitus*	16	0.15	2.69	0.097	n/a	n/a	n/a
*S. planifrons*	16	0.28	4.65	0.025	Distance	− 0.007	0.009
*S. variabilis*	7	0.68	10.87	0.007	Territoriality	0.015	0.010
*S. diencaeus*	13	0.29	4.11	0.041	Distance	− 0.013	0.049
*S. leucostictus*	16	0.17	2.90	0.08	Territoriality	0.013	0.035

**Table 5:** Correlation results of bouts per minute (bpm) and chase rate (left) and bpm and distance traveled (right) for each of the six study species

Species	df	Chase rate			Distance traveled		
		r	p	Correlation strength	*r*	*p*	Correlation strength
*S. partitus*	17	0.53	0.021	High	− 0.34	0.152	Medium
*S. planifrons*	17	0.16	0.526	Low	− 0.6	0.006	High
*S. variabilis*	8	0.79	0.007	Very high	− 0.56	0.094	High
*S. adustus*	16	0.17	0.496	Low	− 0.67	0.003	High
*S. diencaeus*	14	0.45	0.081	Medium	− 0.44	0.087	Medium
*S. leucostictus*	17	0.42	0.07	Medium	− 0.19	0.449	Low

### Species-specific variation in habitat and biological characteristics

Within *Stegastes* territories, benthic cover varied by species depending on the substrate. The Bonferroni post hoc tests revealed that *S. leucostictus* territories were the most different from the other *Stegastes* species due to having the lowest live coral cover and the highest cover of both rubble and sand within their territories than the other five species (Table S3). The Bonferroni post hoc tests revealed that *S. diencaeus* and *S. variabilis* had significantly different boulder cover from other *Stegastes* species due to having the two highest percentages of boulder cover within their territories when compared to the other four species (Table S3).

The average travel speed during spawning was statistically different among species (*F* = 11.15, *p* = < 0.001) and was positively correlated with body size (*r* (80) = 0.49, *p* < 0.001). The post hoc test revealed that the two largest species (*S. diencaeus and S. planifrons*) were significantly faster than the three smallest species (*S. partitus, S. leucostictus, and S. variabilis*) and *S. leucostictus* was significantly slower from the third largest (*S. adustus*) (Table S4).

There was a significant difference in bouts per minute among species (*F* = 21.27, *p* < 0.001). The post hoc test showed trends of larger fish having more spawning bouts than smaller fish and that *S. adustus* had more bouts than all other species in this study. (Table S5). There was also a significant difference in territoriality among species (*F* = 12.36, *p* < 0.001). The post hoc test revealed that territoriality for *S. adustus* was significantly higher than all other species (Table S6). Distance females traveled to spawn also differed significantly among species (*F* = 4.28, *p* = 0.001). The post hoc test showed that *S. variabilis* and *S. diencaeus* traveled significantly further than *S. partitus* and that *S. diencaeus* also traveled further than *S. leucostictus* (Table S7). There was also a significant difference in female body size among species with *S. partitus* and *S. leucostictus* being significantly smaller than the other four species in this study (*F* = 77.43, *p* < 0.001) (Table S8).

There was no correlation between territoriality and travel distance across all *Stegastes* species (Pearson correlation: *r* (99) = − 0.02, *p* = 0.827). Within species, *S. planifrons* was the only species that showed a correlation between territoriality and distance: *S. planifrons* (*r*(17) = − 0.46, *p* = 0.048), *S. adustus* (*r*(16) = − 0.12, *p* = 0.622), *S. diencaeus* (*r*(14) = 0.04, *p* = 0.884), *S. leucostictus* (*r*(17) = − 0.01, *p* = 0.976), *S. partitus* (*r*(17) = − 0.1, *p* = 0.697), and *S. variabilis* (*r*(8) = − 0.45, *p* = 0.194) (Fig S9).

**Fig. 5:**
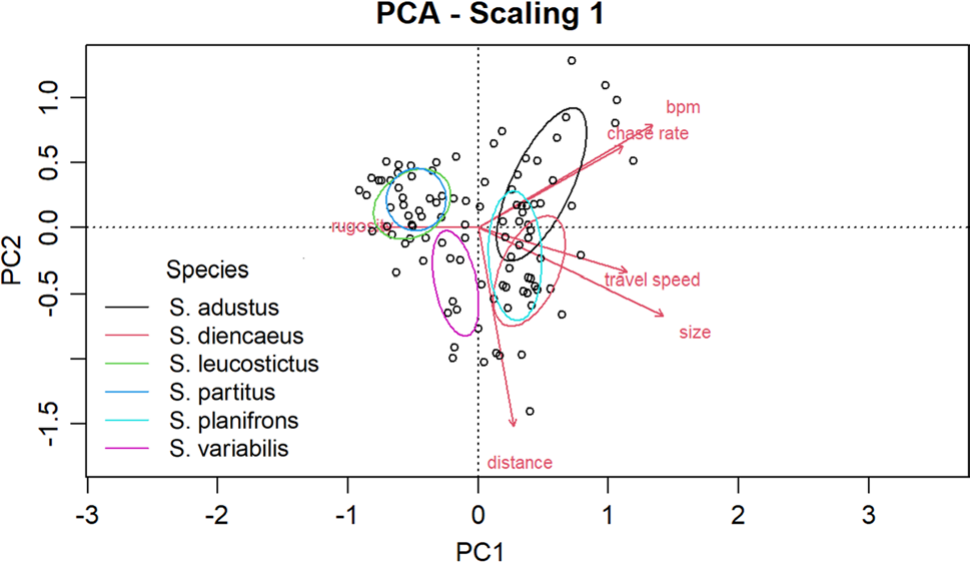
Principal component analysis biplot showing samples (dots) and variable loadings (red vector lines). Ellipses are color-coded to represent species (N for each species as follows: S. adustus = 18; S. diencaeus = 16; S. leucostictus = 19; S. partitus = 19; S. planifrons = 19; S. variabilis = 10)

The PCA revealed groupings among variables in multivariate space. The first and second principal components accounted for 61% of the variability in the dataset, suggesting that the PCA visualization is an effective depiction of the multivariate associations. Ellipses *for S. adustus, S. planifrons*, and *S. diencaeus* grouped together and were characterized by higher travel speed, size, chase rates, and bpm (Fig. 5). Ellipses for *S. partitus* and *S. leucostictus* are also grouped together and were characterized by lower rugosity (Fig. 5). *S. variabilis* clustered on its own (Fig. 5). Phylogenetically, *S. partitus* and *S. planifrons* are closely related, as are *S. leucostictus* and *S. adustus* (Tang et al. 2021). Thus, variation among species does not appear attributable to phylogenetic differences/relatedness.

There was a negative correlation between spawning time and number of defensive attacks during spawning for all study species *r* (555) = − 0.13, *p* = 0.002 (Fig. S6). While not all were significant, species-specific correlations were also all negatively related: *S. leucostictus* (*r*(12) = − 0.35, *p* = 0.224), *S. variabilis* (*r*(15) = − 0.13, *p* = 0.622), *S. partitus* (*r*(37) = − 0.15, *p* = 0.375), *S. diencaeus* (*r*(84) = − 0.29, *p* = 0.007), *S. planifrons* (*r*(115) = − 0.31, *p* = 0.001), and *S. adustus* (*r*(281) = − 0.01, *p* = 0.854).

## Discussion

Studies on central-place activities in ecological systems focus mostly on foraging tactics in tetrapods. Territorial fishes, in which females must leave their territories to spawn with males, present an alternative model system for the study of central-place decisions in animals. This study examined central-place spawning tactics of multiple species of territorial fishes in which females lay a clutch of eggs with a single male that can be completed in either a single event or divided into multiple, shorter, bouts, separated by revisits to the territory. We considered both the costs of absence from the central place and the costs of travel between the territory and the male nest. We found that across six species of *Stegastes* damselfish in the tropical western Atlantic, females appeared sensitive to both intruder pressure and travel distance. Intruder pressure increases the cost of being away from the territory while spawning and is associated with more frequent spawning bouts and thus returns to the home territory. Increased travel distance between male and female territories increases the cost of individual spawning bouts and is associated with less frequent spawning bouts. There were differences in spawning behavior among species that were not attributable to phylogenetic differences within the genus, and that provide further insight into the factors that may influence female spawning decisions. Our findings are consistent with the hypothesis that spawning tactics are driven by habitat, individual, or spawning event-related variation in the costs associated with absence from the central place, and travel.

### Territoriality

Independent of energetic costs of travel, leaving the territory to spawn incurs the potential costs of food loss and eviction of con-and/or heterospecific intruders for territorial damselfishes. In an experimental field study, Sikkel and Kramer (2006) showed that for a large species of tropical Atlantic damselfish (*Microspathodon chrysurus*), dividing spawning into bouts separated by returns to the female’s territory was an effective means of reducing overall intruder pressure and the cost of evicting intruders during the spawning period.

Limiting activity to specific times of the day as a way of minimizing certain costs has been seen in a variety of central-place foragers (Bell 1990; Ruf and Fiedler 2002; Friedlaender et al. 2016). Some caterpillar species forage nocturnally to reduce predation risk despite lower temperatures which are less favorable for digestion and foraging success (Ruf and Fiedler 2002). Baleen whales will rest during the day and forage at night when krill are closer to the surface and more accessible, optimizing their oxygen uses and recovery times (Friedlaender et al 2016). In the tropics, territorial herbivorous damselfish spawn at dawn. This has been hypothesized to reduce intruder pressure, while the female is away spawning, since the primary intruders are herbivorous con- and hetero-specifics whose feeding activity increases during the day (Kohda 1988; Zemke-White et al. 2002; Sikkel et al. 2005). If spawning tactics are responsive to intruder pressure, we expect spawning bouts to become shorter in duration as the sun rises and to be positively related to species and individual-level intruder pressure. Our findings were consistent with both predictions.

Intruder pressure influences behavior in territorial individuals across animal taxa, including birds (Davies and Houston 1983; Temeles 1987; Eberhard and Ewald 1994; Hill et al. 2018; Pavan et al. 2020), mammals (Boutin and Schweiger 1988, McComb et al. 1994, Mayer et al. 2020), and fishes (Warner and Hoffman 1980; Dill et al. 1981; Hixon 1981; Aires et al. 2015; Goncalves and Radford 2022). Most of the work on this topic focuses on adjustments in territory sizes and defense strategies in response to intruder pressure. However, Warner and Hoffman (1980) found that for reef fish species that prioritize territory defense, more energy was devoted to defense in areas with high population density (and thereby higher intruder pressure), detracting from allocations of energy to reproduction. Our results also align with those from the limited existing literature on damselfish spawning behavior across varying levels of intruder pressure. Sikkel and Kramer (2006) found that female *M. chrysurus* that made single spawning trips had lower intruder pressure than those that made multiple trips. For temperate *Hypsypops rubicundus* that spawn throughout the day, mate-searching females with higher intruder pressure made shorter, more frequent mate-searching and spawning bouts (Sikkel 1998).

A variety of time-related indicators are used to influence central-place decisions. For example, Dukas and Real (1993) found that bumble bees use the recent same-day information gathered while foraging to inform them on subsequent foraging decisions. Other species such as social insects or rats use long-term memory (e.g., visual landmarks or memorized routes) to influence decisions (Shettleworth 2009). In this study, intruder pressure was inferred from timed observations of female territorial behavior (i.e., chase rates). While we measured chase rates during spawning and on the day after spawning, it is unclear over what time period individuals integrate their estimates to inform bout durations, either from previous days’ observations or real-time intruder pressure rates.

As with many central-place foragers, intruder pressure appears to contribute to bout allocation for all *Stegastes* species included in this study. Counterintuitively, the two species (*S. variabilis* and *S. leucostictus*) in which territoriality had a significant effect on spawning bout rates had relatively low average chase rates. *S. partitus* also showed a similar trend with territoriality showing a non-significant effect on bouts and overall low chase rates. This suggests that overall intruder pressure and perceived resource value were lower for these species, such that some may have occupied only weakly or partially defended home ranges. Diet for two of the three species consists primarily of small benthic invertebrates (*S. leucostictus)* or zooplankton (*S. partitus*), with the third species (*S. variabilis*) consuming an approximately equal mix of invertebrates and algae (Randall 1967; Nemeth 1997). The emphasis on resource defense in these species may be driven more by shelter rather than safeguarding food from roving herbivores, leading to low chase rates but a heightened emphasis on defense against conspecifics. Thus, similar to predictions from CPF theory, damselfish spawning tactics suggest that species and individuals are sensitive to costs of intruder pressure on the central place while away.

### Travel distance

Most studies on central-place activities also focus on travel distance and associated costs of travel as drivers of decisions regarding travel bout duration and frequency. In CPF theory, longer distances increase travel costs, which is expected to affect the frequency and duration of foraging trips (Olsson et al. 2008). Thus, it is not surprising that travel distance was shown to be negatively related to bout frequency across species in this study. There was no significant association between chase rates and distance to the male nest for five of the six species, suggesting that damselfish with higher intrusion rates are not choosing mates based on proximity but rather based on quality and availability which has been previously observed in *S. partitus* (Knapp and Kovach 1991). Female mate choice in these species is determined the day before spawning, with females swimming directly to the chosen male at first light. Given that mate assessment also involves travel and time away from the territory, females are likely also sensitive to intruder pressure and travel distance during this process, as has been shown for Garibaldi damselfish *H. rubicundus* in temperate reefs (Sikkel 1998).

While the energetic cost of travel increases in proportion to travel distance, other factors can compound this cost. The most likely variable costs in this system are predation risk and attacks by other damselfish (Williams 1978; Karino and Kuwamura 1997). Variation in reproductive tactics in response to predation has been studied in a variety of fish (Robertson and Hofman 1977, Lima and Dill 1990; Hastings 1991; Magnhagen 1991; Chivers et al. 1995). Travel costs associated with predation risk can vary across diel time periods, habitat structures, and species characteristics. In coral-reef environments, predators have the advantage over prey during the crepuscular period (Helfman 1986; Sancho et al. 2000a, 2000b; Campanella et al. 2019). Given that all species included in this study spawn at dawn, diel effects of predation risk should be similar across species. Two obvious variables that could contribute to variation in predation risk-related travel costs in this study are body size and habitat complexity. Smaller damselfish have shown a stronger avoidance of predators than larger damselfish (Helfman 1989; Gauff et al. 2018; Fakan et al. 2024). Furthermore, aggressive intraspecific encounters tend to favor the larger individual in a variety of reef fish species (Shulman 1985; Bonin et al. 2015, Silvia et al. 2020). More complex areas of reef have more places for predators and conspecifics to hide (Quadros et al. 2019) and smaller damselfish species such as *S. partitus* experience higher mortality rates in higher rugosity substrates (Nemeth 1998). Therefore, smaller individuals are likely more vulnerable to attacks by both predators and conspecifics and experience an increase in predation risk with an increase in habitat complexity. Because increasing daylight makes it easier for territorial damselfish to detect intruders and predators, it is expected that chase risk and predation risk change in opposite directions with increasing daylight during the spawning period.

The strength of the relationship between distance traveled and frequency of spawning bouts varied by species. Again, drawing from CPF theory, individuals face tradeoffs between the amount or potential quality of a resource (food) and predation risk that increase with travel distance. Territorial damselfish face a similar predicament as the farther they travel the more likely they are to encounter and thus spawn with a high-quality mate, but at increased risk of predation or injury from conspecifics. In this system, the variation seen among species may be explained, in part, by differences in species-specific habitat and body size. The three species with the highest bouts per minute (bpm), chase rates, and sizes (*S. adustus, S. diencaeus,* and *S. planifrons*) also occupy the most rugose habitat. By contrast, the three species with the lowest bpm, chase rates, and sizes (*S. partitus, S. variabilis, and S. leucostictus*) occupy the least rugose habitat. Habitat has been seen to influence female spawning decisions and reproductive success in *S. leucostictus* with females preferring males with more open spawning sites (Itzkowitz and Makie 1986) as well as in *S. planifrons* where centrally located males received more egg clutches (Meadows 2001). Additionally, areas with higher reef complexity are more rugose and have an overall higher abundance and diversity of fish species (Luckhurst and Luckhurst 1978, Graham and Nash 2012; Santoso et al. 2022), and territorial fish in rugose reefs have been seen to have a higher propensity to take risks than their counterparts in flatter areas of reef (Quadros et al. 2019). Habitat structure has been seen to affect travel decisions for central-place foragers as well (Robinson and Holmes 1982; Benkwitt 2016) showing the parallels between traveling for foraging vs traveling for spawning. While more data are needed to determine specific effects, these results illuminate the importance of habitat composition in spawning tactics by permanently territorial damselfishes.

In addition to adjusting travel distance, animals can adopt travel routes that reduce overall costs, even if overall travel distance is lengthened. The smaller species in this study appeared to choose travel routes that avoided areas that had high densities of conspecific territories despite it lengthening their travel distance (T Hobbs, personal observation). This variation in travel routes by smaller individuals to avoid conspecifics is a tactic seen in central-place foragers like red squirrels (Bakker and Van Vuren 2004). Because conspecific attacks pose a high risk for smaller individuals, varying travel routes to avoid conspecific territories can reduce travel costs. The three larger species, *S. diencaeus*, *S. adustus*, and *S. planifrons,* had the fastest travel speeds on average and were very deliberate in their travel. They appeared to go directly from female to male territories with less stopping and perceived caution when compared to the other three smaller species (personal observation, T Hobbs). Intermittent locomotion and slower speeds can decrease an individual’s chance of being detected (Halsey 2016), and larger individuals have a lower cost associated with travel when compared to smaller fish (Wakeman and Wohlschlag 1981), which are likely reasons for the slower, more cautious travel tactic taken by smaller damselfish individuals.

### Opportunity costs

Central place foraging and other central-place activities may also involve opportunity costs. While foraging opportunities may seem to be the most likely such costs in this system, relatively little feeding, which peaks in the afternoon, occurs during the dawn-spawning period in herbivorous damselfishes (Zemke-White et al 2002; Sikkel et al. 2005; Khait et al. 2013). However, we have observed conspecifics that intrude on female territories during her absence feeding at accelerated rates (T. Hobbs and P. Sikkel pers obs), suggesting an attempt to “steal” food. In contrast, infestation with parasitic gnathiid isopods and interactions with cleaners that remove them peak at dawn, during the spawning period (Sikkel et al. 2005, 2006). Thus, missed cleaning opportunities would be the most likely opportunity cost. This would apply to larger species such as *S. diencaeus* that both have higher ectoparasite loads and interact frequently with cleaners (Cheney and Cote 2001, Sikkel et al. 2006). Indeed, in the largest territorial damselfish in the Caribbean (*Microspathodon chrysurus*), females interrupt spawning to visit cleaning stations and interact with cleaners during returns to their territory over the spawning period (Sikkel et al. 2005). Cleaner fishes and shrimps were rare at our study sites and we did not observe such behavior in *Stegastes* in this study.

Finally, one potential opportunity cost for females leaving the nest while spawning could be loss of access to their chosen nest due to competition from other females. While nests of male damselfishes can and typically do hold multiple clutches of eggs, males or females may only allow one female at a given time in the nest. In *M. chrysurus*, females will chase other approaching females from the nest in which they are visiting or spawning. However, this does not happen in Caribbean *Stegastes*, where females (and males) tolerate more than one female in the nest at a given time. Thus, there seems to be no risk of loss of access to the male nest if females leave to return to their territory.

### Further comparisons with CPF theory

Considering the broader similarities and differences of our system with classic CPF systems upon which central-place theory is based, the most direct parallels with respect to potential costs associated with time away from the territory (central place) and with travel between the central place and the external resource. General predictions from theory for how increases in these costs should impact decisions (Andersson 1981; Boutin and Schweiger 1988; Bell 1990) were supported. Where our system deviates is with respect to the variation in travel cost in both space and time, and the benefits of being away from the central place. Rather than accumulate energy resources as central-place foragers do, female damselfish deposit eggs in male nests which are then cared for by the male. The total time required for egg laying should be a simple function of the number of eggs a female has to lay (hundreds to thousands) on a given morning, with benefits of time away from the territory increasing with the number laid and their probability of hatching, and possibly also genetic benefits associated with male quality. For females of some permanently territorial species of temperate damselfish, mate-searching and spawning occur throughout the day, with central-place mate searching leading to central-place spawning on the same day (Sikkel 1998). However, in tropical species like those studied here, mate choice occurs during the day prior and so is temporally decoupled from the spawning event itself. Thus, for a given female on a given spawning day, there is only one available “patch” (nest) and the benefits it yields per unit time are “known” and fixed, and there is no depletion of resources in the “patch” (nest) over time, although there are fewer eggs to be laid. However, spawning site quality can vary among females on a given day or within females among days, and females may be more willing to incur higher travel and/or intruder costs to spawn in higher quality nests or with higher quality males. Incorporating mate/nest quality as part of the benefit of being away from the central place and its impact on female egg-laying tactics is clearly an avenue for future study. Indeed, travel costs and costs associated with absence from the territory could impose limits on how far females will travel to spawn with a male and can be both cause and consequence of landscape-level structuring of *Stegastes* populations (e.g., Lima and Zolner 1996).

Finally, this work highlights an opportunity to broaden and refine the existing CPF theoretical framework by introducing different central-place activities that include different specific costs and benefits. It also positions territorial damselfish as a valuable model system for investigating these dynamics and empirically testing theoretical predictions.

## Supplementary Information

Below is the link to the electronic supplementary material.

Supplementary file1 (DOCX 2820 KB)

## Data Availability

Not applicable.
