# Supplementary material for: The long and short of it: travel distance and territorial intruder pressure predict central-place spawning tactics among Caribbean Stegastes damselfishes
Source: Oecologia. 2025 Jul 8;207(8):127. doi: 10.1007/s00442-025-05761-w (PMC12238210; doi:10.1007/s00442-025-05761-w)
Supplement: Supplementary file 1 — Supplementary file1 (DOCX 2820 KB) [file 442_2025_5761_MOESM1_ESM.docx]

The long and short of it: Travel distance and territorial intruder pressure predict central-place spawning tactics among Caribbean *Stegastes* damselfishes

**Authors:** Taylor L. Hobbs^1^, Richard S. Nemeth^1^, Donna Nemeth^1^, Kayla M. Blincow^1^, Paul C. Sikkel^1,2*^

^1^University of the Virgin Islands, Center for Marine and Environmental Studies, St. Thomas, United States Virgin Islands, 00802, USA

^2^Department of Marine Biology and Ecology, Rosenstiel School of Marine, Atmospheric and Earth Science, University of Miami, Miami, FL, USA

*Corresponding author:

E-mail: [pcs75@earth.miami.edu](mailto:pcs75@earth.miami.edu)

Tel: +1 (270) 293-5489

Fax: +1 (305) 421-4600

**Supplemental data**

**Figures**


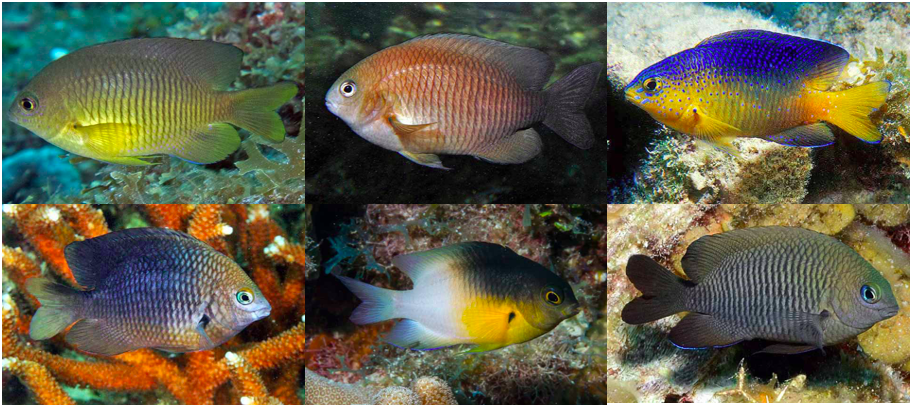


**Fig. S1** Adults of six *Stegastes* species found in the USVI. Photos adapted from Smithsonian Tropical Research Institute (2015). In order from left to right; (top) *S. variabilis* (Smith, n.d.), *S. adustus* (Robertson, n.d.), *S. leucostictus* (Robertson, n.d.), (bottom) *S. planifrons* (Bryant, n.d.), *S. partitus* (Garin, n.d.), *S. diencaeus* (Graham, n.d.)


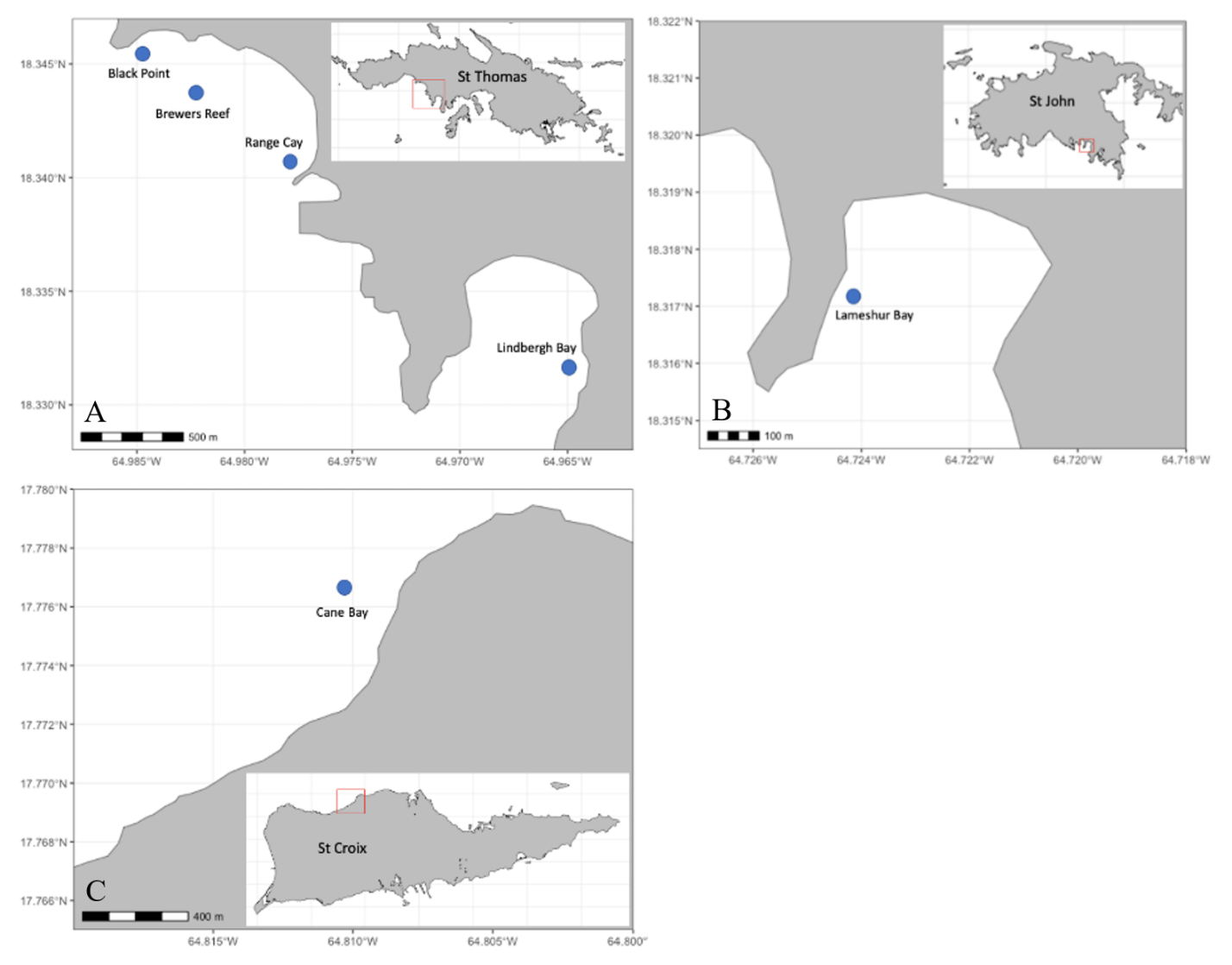


**Fig. S2** Map of study sites (blue dots) on St Thomas (panel A), St. John (panel B), and St. Croix (panel C).


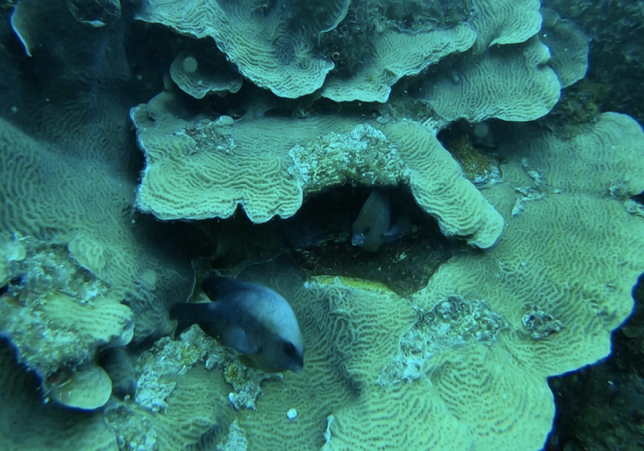

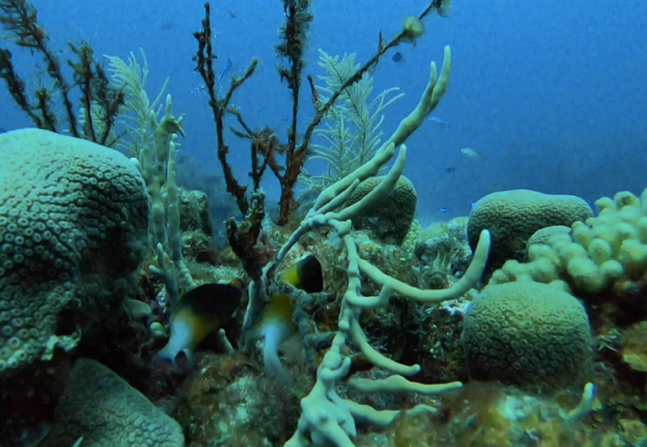


**Fig. S3** Each photo shows a mating pair of *S. planifrons* (left) and *S. partitus* (right) in a nest within the male’s territory. In both photos the male is located on the left-hand side and the female is located on the right. Photos: T. Hobbs


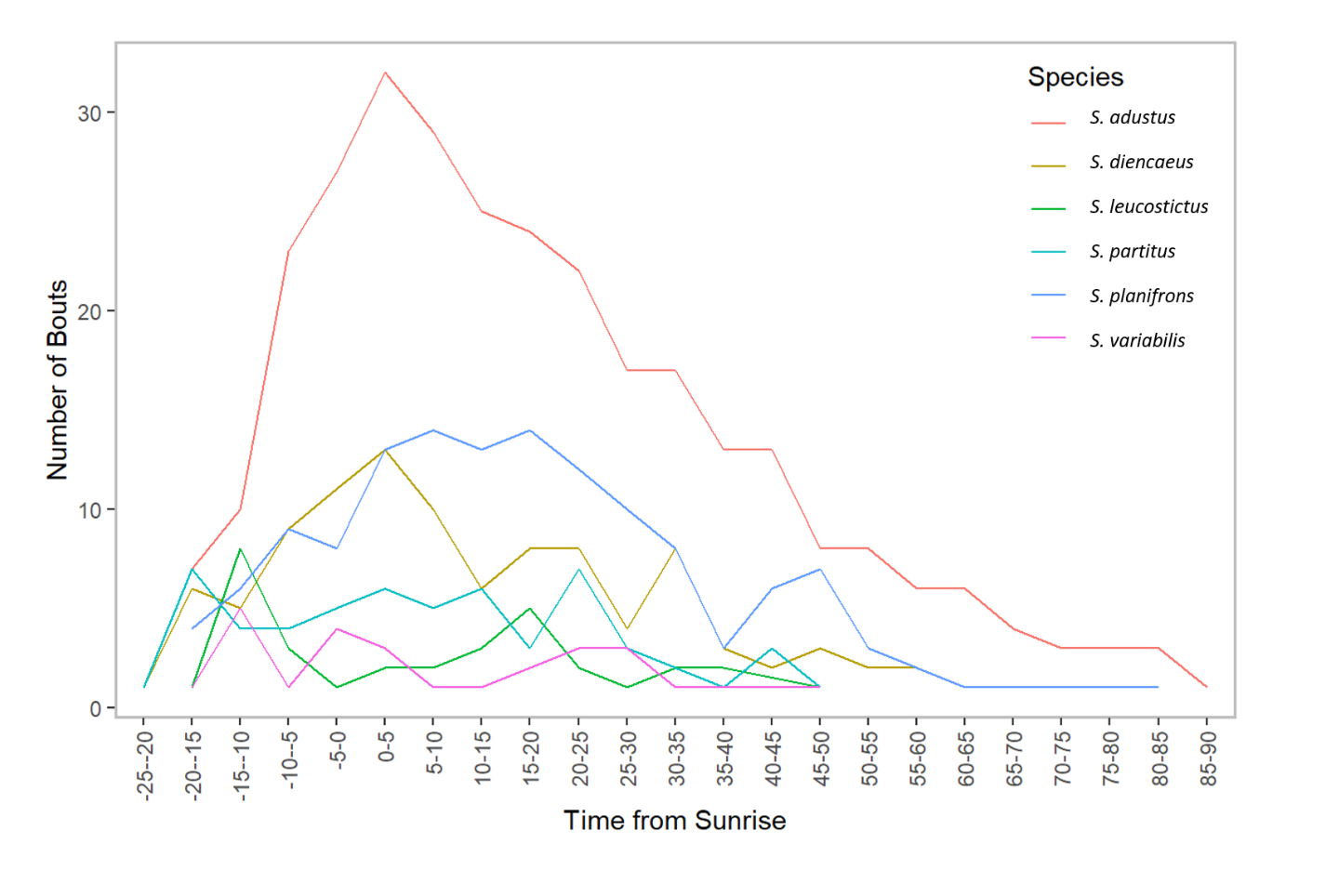


**Fig. S4** Number of spawning bouts by time relative to sunrise binned in 5-minute intervals. Time intervals are depicted relative to time from sunrise (0).


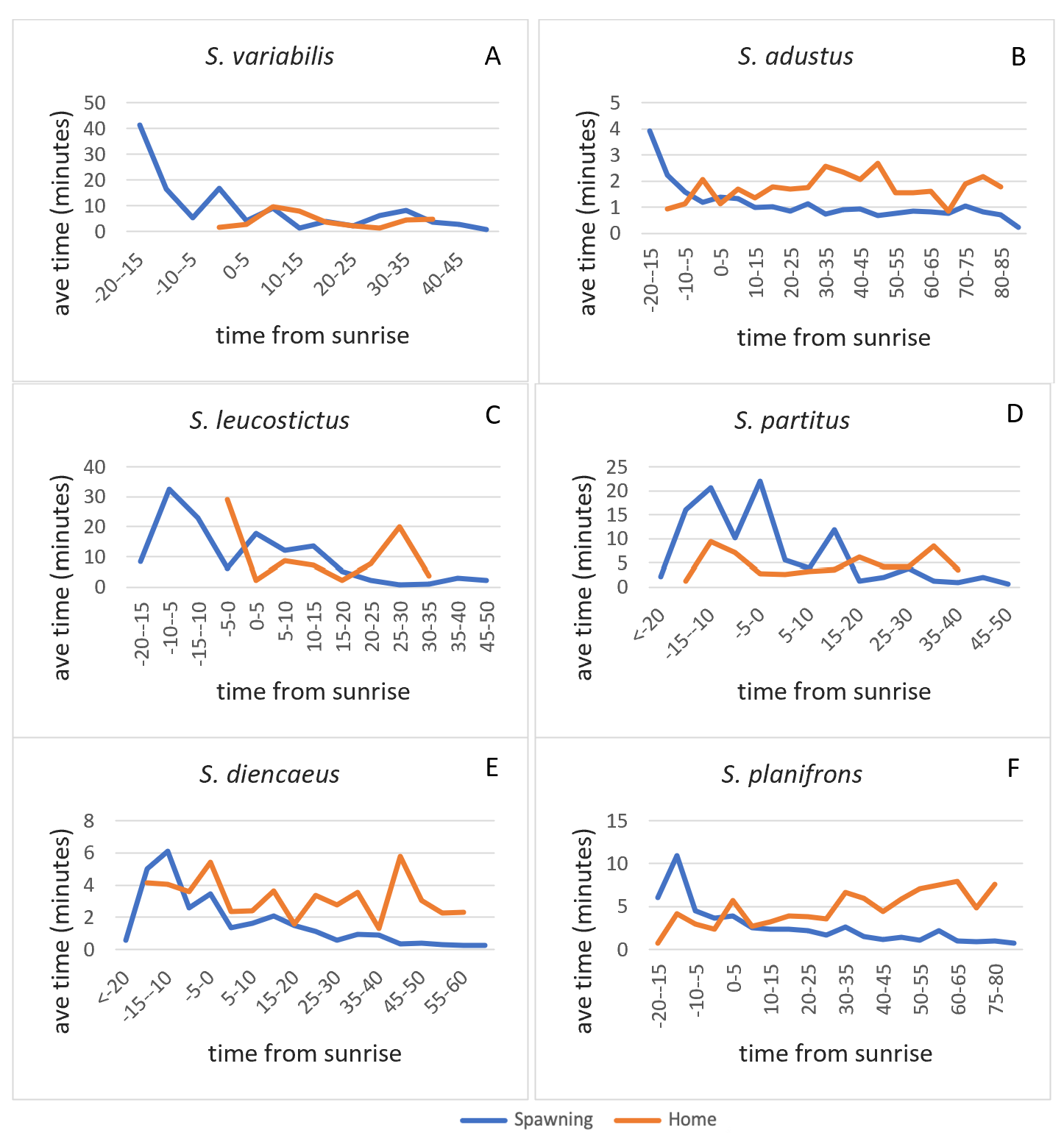


**Fig. S5** Average spawning and home times by species (A - F) as a function of time from sunrise throughout the spawning session.

spawning time (seconds)

chase rate

chase rate


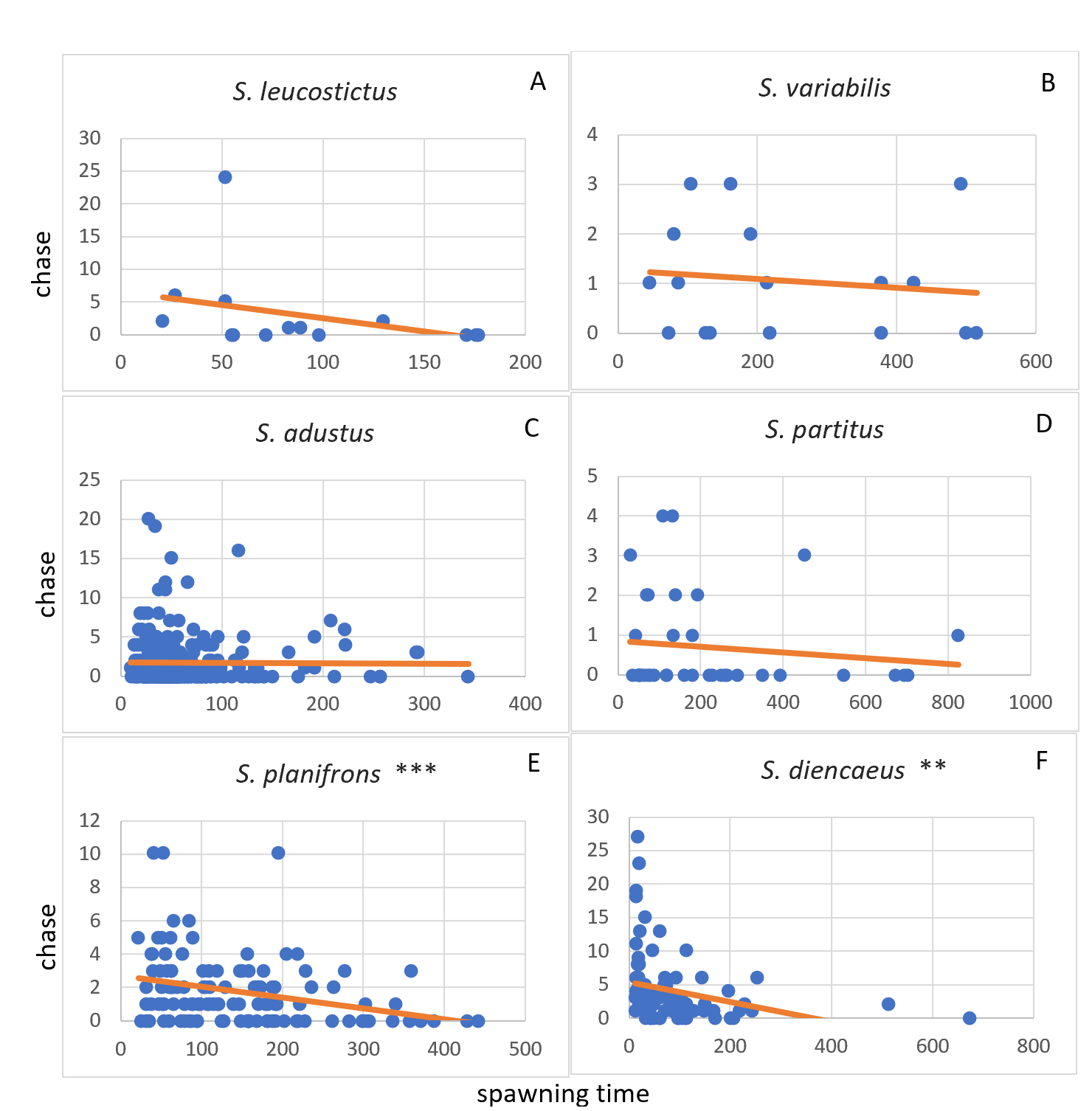


**Fig. S6**. Relationships between time spent spawning and defensive attacks during spawning showing raw data (blue dots) trend lines (orange lines), and significance (asterisks) for each species (A – F).

**Tables**

**Table S1** Number of females observed for each species at six study sites (n=101).

|  | *S. variabilis* | *S. diencaeus* | *S. adustus* | *S. planifrons* | *S. partitus* | *S. leucostictus* | Total |
| --- | --- | --- | --- | --- | --- | --- | --- |
| Range Cay | 2 | 3 | 5 | 6 | 6 | 11 | 33 |
| Black Point | 1 | 4 | 2 | 0 | 2 | 6 | 15 |
| Brewers Reef | 0 | 3 | 0 | 8 | 2 | 0 | 13 |
| Lindbergh Bay | 6 | 0 | 10 | 0 | 0 | 2 | 18 |
| Lameshur Bay | 1 | 4 | 1 | 3 | 0 | 0 | 9 |
| Cane Bay | 0 | 2 | 0 | 2 | 9 | 0 | 13 |
| Total | 10 | 16 | 18 | 19 | 19 | 19 | 101 |

**Table S2** Biological, dependent, and environmental variables showing mean, median, standard deviation, range, and confidence intervals for each species.

|  | **Mean** | **Median** | **SD** | **Range** | **95% CI lower limit** | **95% CI upper limit** |
| --- | --- | --- | --- | --- | --- | --- |
| **Travel distance between territories (m)** |  |  |  |  |  |  |
| S. planifrons | 4.54 | 3 | 4.55 | 15 | 2.49 | 6.59 |
| S. partitus | 2.21 | 2 | 2.02 | 5.9 | 1.3 | 3.12 |
| S. leucostictus | 3.02 | 3.2 | 2.51 | 8.2 | 1.89 | 4.15 |
| S. adustus | 4.12 | 2.4 | 4.28 | 13.2 | 2.14 | 6.1 |
| S. diencaeus | 6.76 | 6.5 | 3.47 | 13 | 5.06 | 8.46 |
| S. variabilis | 6.55 | 6.8 | 3.62 | 10.5 | 4.31 | 8.79 |
| **Chase rates (chases/hr^-1^)** |  |  |  |  |  |  |
| S. planifrons | 15.47 | 15 | 6.11 | 25 | 12.72 | 18.22 |
| S. partitus | 8.11 | 8 | 7.72 | 33 | 4.63 | 11.58 |
| S. leucostictus | 14.47 | 8 | 19.43 | 82 | 5.74 | 23.21 |
| S. adustus | 39.17 | 34.5 | 20.56 | 88 | 29.67 | 48.67 |
| S. diencaeus | 24.19 | 18.5 | 15.36 | 53 | 16.66 | 31.72 |
| S. variabilis | 4.9 | 5 | 3.18 | 8 | 2.93 | 6.87 |
| **Female Body Size (mm)** |  |  |  |  |  |  |
| S. planifrons | 80.42 | 82 | 9.15 | 40 | 76.31 | 84.53 |
| S. partitus | 49.11 | 46 | 8.01 | 27 | 45.5 | 52.71 |
| S. leucostictus | 48.05 | 46 | 6.89 | 27 | 44.95 | 51.15 |
| S. adustus | 74.83 | 76.5 | 9.12 | 30 | 70.62 | 79.05 |
| S. diencaeus | 86.06 | 85.5 | 5.59 | 22 | 83.32 | 88.8 |
| S. variabilis | 74.2 | 76 | 6.81 | 22 | 69.98 | 78.42 |
| **Spawning bouts (bouts/ min^-1^)** |  |  |  |  |  |  |
| S. planifrons | 0.15 | 0.15 | 0.05 | 0.21 | 0.13 | 0.18 |
| S. partitus | 0.08 | 0.07 | 0.04 | 0.14 | 0.06 | 0.1 |
| S. leucostictus | 0.06 | 0.05 | 0.04 | 0.14 | 0.05 | 0.08 |
| S. adustus | 0.3 | 0.27 | 0.15 | 0.56 | 0.23 | 0.37 |
| S. diencaeus | 0.18 | 0.14 | 0.1 | 0.34 | 0.13 | 0.23 |
| S. variabilis | 0.08 | 0.08 | 0.04 | 0.11 | 0.06 | 0.1 |
| **Rugosity** |  |  |  |  |  |  |
| S. planifrons | 0.72 | 0.75 | 0.11 | 0.4 | 0.67 | 0.77 |
| S. partitus | 0.79 | 0.81 | 0.09 | 0.32 | 0.75 | 0.83 |
| S. leucostictus | 0.81 | 0.82 | 0.08 | 0.28 | 0.77 | 0.85 |
| S. adustus | 0.74 | 0.76 | 0.1 | 0.43 | 0.69 | 0.79 |
| S. diencaeus | 0.75 | 0.76 | 0.09 | 0.3 | 0.71 | 0.8 |
| S. variabilis | 0.76 | 0.75 | 0.06 | 0.17 | 0.72 | 0.8 |

**Table S3** Post-hoc results showing differences in benthic cover by species with all non-significant results removed.

| **Live Coral** |  | **Mean diff.** | **Std. Error** | **t** | **p** | **95% CI lower limit** | **95% CI upper limit** |
| --- | --- | --- | --- | --- | --- | --- | --- |
| S. leucostictus | S. adustus | 17.66 | 5.046 | 3.5 | 0.01 | 2.08 | 33.24 |
| S. diencaeus | S. partitus | -16.96 | 5.205 | -3.26 | 0.023 | -33.03 | -0.89 |
| S. leucostictus | S. partitus | -20 | 4.977 | -4.02 | 0.002 | -35.37 | -4.63 |
| S. leucostictus | S. planifrons | -18.16 | 4.977 | -3.65 | 0.007 | -33.52 | -2.79 |
| **Boulder** |  |  |  |  |  |  |  |
| S. diencaeus | S. adustus | -39.37 | 5.291 | -7.44 | <0.001 | -55.71 | -23.04 |
| S. diencaeus | S. leucostictus | 39.38 | 5.225 | 7.54 | <0.001 | 23.24 | 55.51 |
| S. diencaeus | S. partitus | 39.38 | 5.225 | 7.54 | <0.001 | 23.24 | 55.51 |
| S. diencaeus | S. planifrons | 36.74 | 5.225 | 7.03 | <0.001 | 20.61 | 52.88 |
| S. variabilis | S. adustus | -42.5 | 6.073 | -7 | <0.001 | -61.25 | -23.75 |
| S. variabilis | S. leucostictus | -42.5 | 6.016 | -7.06 | <0.001 | -61.07 | -23.93 |
| S. variabilis | S. partitus | -42.5 | 6.016 | -7.06 | <0.001 | -61.07 | -23.93 |
| S. variabilis | S. planifrons | -39.87 | 6.016 | -6.63 | <0.001 | -58.44 | -21.29 |
| **Sand** |  |  |  |  |  |  |  |
| S. leucostictus | S. adustus | -13.14 | 2.141 | -6.14 | <0.001 | -19.75 | -6.53 |
| S. leucostictus | S. diencaeus | -11.23 | 2.209 | -5.09 | <0.001 | -18.05 | -4.41 |
| S. leucostictus | S. partitus | 9.21 | 2.112 | 4.36 | 0.001 | 2.69 | 15.73 |
| S. leucostictus | S. planifrons | 10.53 | 2.112 | 4.98 | <0.001 | 4 | 17.05 |
| **Rubble** |  |  |  |  |  |  |  |
| S. leucostictus | S. adustus | -37.72 | 3.666 | -10.29 | <0.001 | -49.04 | -26.4 |
| S. leucostictus | S. diencaeus | -39.18 | 3.782 | -10.36 | <0.001 | -50.86 | -27.5 |
| S. leucostictus | S. partitus | 37.37 | 3.616 | 10.33 | <0.001 | 26.2 | 48.53 |
| S. leucostictus | S. planifrons | 38.16 | 3.616 | 10.55 | <0.001 | 26.99 | 49.32 |
| S. leucostictus | S. variabilis | 36.55 | 4.355 | 8.39 | <0.001 | 23.11 | 50 |

**Table S4** Post-hoc results showing differences in travel speed during spawning by species with non-significant results removed.

| **Speed** |  | Mean diff. | Std. Error | t | p | 95% CI lower limit | 95% CI upper limit |
| --- | --- | --- | --- | --- | --- | --- | --- |
| **S. leucostictus** | **S. diencaeus** | -0.29 | 0.061 | -4.71 | <0.001 | -0.47 | -0.1 |
| **S. leucostictus** | **S. planifrons** | -0.35 | 0.06 | -5.78 | <0.001 | -0.53 | -0.16 |
| **S. variabilis** | **S. diencaeus** | -0.22 | 0.071 | -3.14 | 0.036 | -0.45 | 0 |
| **S. variabilis** | **S. planifrons** | -0.28 | 0.07 | -4.02 | 0.002 | -0.5 | -0.06 |
| **S. partitus** | **S. diencaeus** | -0.28 | 0.065 | -4.35 | 0.001 | -0.49 | -0.08 |
| **S. partitus** | **S. planifrons** | -0.34 | 0.065 | -5.33 | <0.001 | -0.54 | -0.14 |

**Table S5** Post hoc results comparing bpm among species with non-significant results removed.

|  |  | Mean diff. | Std. Error | p | 95% CI lower limit | 95% CI upper limit |
| --- | --- | --- | --- | --- | --- | --- |
| S. adustus | **S. variabilis** | 0.22 | 0.032 | <0.001 | 0.12 | 0.32 |
| S. adustus | **S. diencaeus** | 0.12 | 0.028 | 0.001 | 0.03 | 0.21 |
| S. adustus | **S. planifrons** | 0.15 | 0.027 | <0.001 | 0.06 | 0.23 |
| S. adustus | **S. partitus** | 0.22 | 0.027 | <0.001 | 0.14 | 0.3 |
| S. adustus | **S. leucostictus** | 0.24 | 0.027 | <0.001 | 0.15 | 0.32 |
| S. diencaeus | **S. partitus** | 0.1 | 0.028 | 0.007 | 0.02 | 0.19 |
| S. diencaeus | **S. leucostictus** | 0.12 | 0.028 | 0.001 | 0.03 | 0.2 |
| S. leucostictus | **S. planifrons** | -0.09 | 0.026 | 0.016 | -0.17 | -0.01 |

**Table S6** Post hoc results comparing the chase rates of females among species with non-significant results removed.

|  |  | Mean diff. | Std. Error | p | 95% CI lower limit | 95% CI upper limit |
| --- | --- | --- | --- | --- | --- | --- |
| S. adustus | **S. variabilis** | 34.27 | 5.63 | <0.001 | 16.88 | 51.65 |
| S. adustus | **S. planifrons** | 23.69 | 4.696 | <0.001 | 9.19 | 38.19 |
| S. adustus | **S. partitus** | 31.06 | 4.696 | <0.001 | 16.56 | 45.56 |
| S. adustus | **S. leucostictus** | 24.69 | 4.696 | <0.001 | 10.19 | 39.19 |
| S. adustus | **S. diencaeus** | 14.98 | 4.905 | 0.044 | -0.17 | 30.12 |
| S. diencaeus | **S. variabilis** | 19.29 | 5.755 | 0.017 | 1.52 | 37.06 |
| S. diencaeus | **S. partitus** | 16.08 | 4.844 | 0.019 | 1.13 | 31.04 |

**Table S7** Post hoc results comparing the distance females traveled to mate among species with non-significant results removed.

|  |  | Mean diff. | Std. Error | P | 95% CI lower limit | 95% CI upper limit |
| --- | --- | --- | --- | --- | --- | --- |
| S. variabilis | **S. partitus** | -4.34 | 1.371 | 0.031 | -8.57 | -0.11 |
| S. diencaeus | **S. partitus** | 4.55 | 1.191 | 0.004 | 0.88 | 8.23 |
| S. diencaeus | **S. leucostictus** | 3.74 | 1.191 | 0.034 | 0.06 | 7.42 |

**Table S8** Post hoc results comparing the female’s body size among species with non-significant results removed.

|  |  | **Mean diff.** | **Std. Error** | **p** | **95% CI lower limit** | **95% CI upper limit** |
| --- | --- | --- | --- | --- | --- | --- |
| **S. adustus** | **S. diencaeus** | -11.23 | 2.689 | 0.001 | -19.53 | -2.93 |
| **S. adustus** | **S. partitus** | 25.73 | 2.574 | <0.001 | 17.78 | 33.68 |
| **S. adustus** | **S. leucostictus** | 26.78 | 2.574 | <0.001 | 18.83 | 34.73 |
| **S. variabilis** | **S. diencaeus** | -11.86 | 3.155 | 0.004 | -21.6 | -2.12 |
| **S. variabilis** | **S. partitus** | 25.09 | 3.057 | <0.001 | 15.65 | 34.53 |
| **S. variabilis** | **S. leucostictus** | 26.15 | 3.057 | <0.001 | 16.71 | 35.59 |
| **S. diencaeus** | **S. partitus** | 36.96 | 2.655 | <0.001 | 28.76 | 45.16 |
| **S. diencaeus** | **S. leucostictus** | 38.01 | 2.655 | <0.001 | 29.81 | 46.21 |
| **S. planifrons** | **S. partitus** | 31.32 | 2.539 | <0.001 | 23.48 | 39.16 |
| **S. planifrons** | **S. leucostictus** | 32.37 | 2.539 | <0.001 | 24.53 | 40.21 |
